# Supplementary material for: Odorant-Binding and Chemosensory Proteins in Anthonomus eugenii (Coleoptera: Curculionidae) and Their Tissue Expression
Source: Int J Mol Sci. 2023 Feb 8;24(4):3406. doi: 10.3390/ijms24043406 (PMC9961831; doi:10.3390/ijms24043406)
Supplement: Supplementary file 1 [file ijms-24-03406-s001.zip › suppl..pdf]

International Journal of *Molecular Sciences*

MDPI

# Supplementary Information for

## ODORANT-BINDING AND CHEMOSENSORY PROTEINS IN *Anthonomus eugenii* (Coleoptera: Curculionidae) AND THEIR TISSUE EXPRESSION

**Pablo Lechuga Paredes[1], Obdulia Lourdes Segura-León [1*], Juan Cibrián-Tovar[1], Brenda Torres-Huerta[1], Julio César Velázquez-González [2] and José Luis Cruz-Jaramillo[3]**

[1] Entomology and Acarology Program, Colegio de Postgraduados Campus Montecillo, Mexico-Texcoco, km. 36.5, C.P. 56230, State of Mexico, Mexico
[2] Koppert de México. Circuito el Marqués norte No. 82. Industrial Park el Marqués, 76246. Querétaro, Mexico
[3] Massive Sequencing Laboratory of Centro Nacional de Referencia Fitosanitaria of SENASICA, Mexico-Pachuca Highway, Km. 37.5 Tecámac, 55740, State of Mexico, Mexico
* Correspondence: sleon@colpos.mx ; Tel.: +52-5540093079

**Table S1.** Resume Illumina MiSeq™ System and assembly of four *A. eugenii* libraries

| Libraries | Media length (bp) | Clean reads | Total Bases | GC% | Q20 | Q30 |
|---|---|---|---|---|---|---|
| Aeug1-1 | 75 | 3.008242 M | 227.087721 M | 35.44 | 97.6 | 91.95 |
| Aeug1-2 | 75 | 2.968824 M | 224.131026 M | 35.33 | 95.07 | 90.21 |
| Aeug2-1 | 75 | 1.729747 M | 130.552430 M | 32.93 | 97.7 | 92.28 |
| Aeug2-2 | 75 | 1.719634 M | 129.805249 M | 32.88 | 94.96 | 89.96 |
| ***De novo* assembly** | **Transcripts** | **Unigenes** | **LM (pb)** | **N30** | **N50** | **GC%** |
| | 7,130 | 5,855 | 330 | 927 | 563 | 45.86 |

**Table S2.** GO Terms analysis resume.

| | Genes | 2194 |
|---|---|---|
| **GO Terms** | Molecular Function | 1774 |
| | Biological Process | 1352 |
| | Cellular Component | 883 |
| | Total Annotated Terms | 4009 |

**Table S3.** Genes annotated per each GO term.

| GO Id | Clasification | Number of transcripts | Percetage | GO term |
|---|---|---|---|---|
| **GO:0044464** | CC | 517 | 23.6 | Cell part |

| | | | | |
|---|---|---|---|---|
| **GO:0005623** | CC | 517 | 23.6 | Cell |
| **GO:0032991** | CC | 341 | 15.5 | Protein-contanining complex |
| **GO:0005576** | CC | 74 | 3.4 | Extracellular region |
| **GO:0044421** | CC | 19 | 0.9 | Extracellular region part |
| **GO:0044422** | CC | 214 | 9.8 | Organelle part |
| **GO:0043226** | CC | 392 | 17.9 | Organelle |
| **GO:0044425** | CC | 332 | 15.1 | Membrane part |
| **GO:0016020** | CC | 453 | 20.6 | Membrane |
| **GO:0031974** | CC | 53 | 2.4 | Membrane-enclosed lumen |
| **GO:0044215** | CC | 9 | 0.4 | Other organism |
| **GO:0044217** | CC | 9 | 0.4 | Other organism part |
| **GO:0099080** | CC | 8 | 0.4 | Supramolecular complex |
| **GO:0045202** | CC | 1 | 0.11 | Synapse |
| **GO:0019012** | CC | 34 | 1.5 | Virion |
| **GO:0044423** | CC | 32 | 1.5 | Virion part |
| **GO:0030054** | CC | 6 | 0.3 | Cell junction |
| **GO:0003824** | MF | 857 | 39.1 | Catalytic activy |
| **GO:0005488** | MF | 1045 | 47.6 | Binding |
| **GO:0016209** | MF | 19 | 0.9 | Antioxidant activity |
| **GO:0140104** | MF | 3 | 0.1 | Molecular carrier activity |
| **GO:0005215** | MF | 175 | 8 | Transporter activity |
| **GO:0060089** | MF | 15 | 0.7 | Molecular transducer activity |
| **GO:0140110** | MF | 43 | 2 | Transcription regulator activity |
| **GO:0005198** | MF | 134 | 6.1 | Structural molecule activity |
| **GO:0098772** | MF | 53 | 2.4 | Molecular function regulator |
| **GO:0038024** | MF | 2 | 0.1 | Cargo receptor activity |
| **GO:0009987** | BP | 825 | 37.6 | Cellular process |
| **GO:0008152** | BP | 908 | 41.4 | Metabolic process |
| **GO:0065007** | BP | 252 | 11.5 | Biological regulation |
| **GO:0051179** | BP | 312 | 14.2 | Localization |
| **GO:0002376** | BP | 12 | 0.5 | Immune system process |
| **GO:0050896** | BP | 153 | 7 | Response to stimulus |
| **GO:0050789** | BP | 214 | 9.8 | Regulation of biological process |
| **GO:0048519** | BP | 20 | 0.9 | Negative regulation of biological process |
| **GO:0023052** | BP | 75 | 3.4 | Signaling |
| **GO:0071840** | BP | 142 | 6.5 | Cellular component organization or biogenesis |
| **GO:0040011** | BP | 23 | 1 | Locomotion |
| **GO:0032502** | BP | 36 | 1.6 | Developmental process |
| **GO:0048518** | BP | 26 | 1.2 | Positive regulation of biological process |
| **GO:0032501** | BP | 47 | 2.1 | Multicellular organismal process |
| **GO:0051704** | BP | 79 | 3.6 | Multi-organims process |
| **GO:0022414** | BP | 18 | 0.8 | Proproductive process |

| | | | | |
|---|---|---|---|---|
| **GO:0000003** | BP | 21 | 1 | Reproduction |
| **GO:0098754** | BP | 1 | 0.07 | Detoxification |
| **GO:0001906** | BP | 1 | 0.07 | Cell killing |
| **GO:0022610** | BP | 25 | 1.1 | Biological adhesion |
| **GO:0008283** | BP | 2 | 0.1 | Cell proliferation |
| **GO:0048511** | BP | 1 | 0.07 | Rhythmic process |

CC: Cellular Component; MF: Molecular Function; BP: Biological Process. **The percentages are related with the total genes annotated in the GO analysis.**

**Table S4.** Results of BLASTx and DescribePROT homology analysis. The table shows the best hits with the highest percentage of identity.

| AeugOBP | OBP Family | Unigen | ORF-C | Protein | Best BLASTx match (UniProtKB database) | | | | | Best DescribePROT match/ UniProtKB | | | | |
|---|---|---|---|---|---|---|---|---|---|---|---|---|---|---|
| | | Size (bp) | (bp) | Size (aa) | Name | Acc. Num | E-value | % Id | Sp. | Name | Acc. Num. | E-value | % Id | Sp. |
| **AeugOBPC01** | Minus-C | 529 | (408) | 136aa | AgraOBP3 | A0A2P9JZE4 | 1.28E-19 | 71.42 | Agra | TcasOBPC01 | D6WS42 | 1.36E-05 | 24% | Tcas |
| **AeugOBPC03** | Minus-C | 748 | (372) | 124aa | AgraOBP3 | A0A2S1KLJ4 | 2.3 E-19 | 60.93 | Agra | TcasOBPC03 | D6WS44 | 0.012 | 26% | Tcas |
| **AeugOBPC04a** | Minus-C | 224 | I (219) | 73aa | AgraOBP12 | A0A2P9JZF1 | 1.51E-28 | 61.42 | Agra | TcasOBPC04 | D6WS38 | 2.23E-07 | 35% | Tcas |
| **AeugOBPC04b** | Minus-C | 554 | (321) | 107aa | AgraOBP22 | A0A0X9F729 | 6.67E-20 | 64.61 | Agra | TcasOBPC04 | D6WS38 | 2.23E-07 | 31% | Tcas |
| **AeugOBPC04c** | Minus-C | 617 | (402) | 134aa | ItypOBP7 | M3VHC0 | 4.52E-59 | 69.92 | Ityp | TcasOBPC04 | D6WS38 | 2.23E-07 | 38% | Tcas |
| **AeugOBPC04d** | Minus-C | 982 | (390) | 130aa | AgraOBP16 | A0A2P9JZF4 | 2.14E-49 | 64.7 | Agra | TcasOBPC04 | D6WS38 | 2.23E-07 | 45% | Tcas |
| **AeugOBP6** | Classic | 613 | 3′ (351) | 117aa | RferOBP23 | A0A2R3ZV81 | 3.33E-36 | 68.46 | Rfer | TcasOBP6 | D6WM82 | 1.49E-41 | 59% | Tcas |
| **AeugOBP9** | Classic | 502 | 5′ (441) | 147aa | DponOBP16 | A0A0H3W5N8 | 5.61E-53 | 53.2 | Dpon | TcasOBP9 | D6X1W1 | 6.78E-48 | 52% | Tcas |
| **AeugOBPC09** | Minus-C | 246 | 5′ (219) | 73aa | AgraOBP3 | A0A2P9JZE4 | 3.42E-23 | 61.64 | Agra | TcasOBPC09 | D6WS40 | 0.000865 | 33% | Tcas |
| **AeugOBPC10a** | Minus-C | 568 | (366) | 122aa | AgraOBP9 | A0A2P9JZE9 | 3.13 E-66 | 84.61 | Agra | TcasOBPC10 | D6X4H5 | 2.6E-07 | 34% | Tcas |
| **AeugOBPC10b** | Minus-C | 745 | (426) | 141aa | AgraOBP13 | A0A2P9JZF2 | 3. E-29 | 49.50 | Agra | TcasOBPC10 | D6X4H5 | 0.12 | 33% | Tcas |
| **AeugOBP10** | Classic | 742 | (450) | 150aa | AgraOBP5 | A0A2P9JZE5 | 1.95E-81 | 87.97 | Agra | TcasOBP10 | D6WS37 | 8.68E-39 | 56% | Tcas |
| **AeugOBPC13** | Minus-C | 535 | (435) | 145aa | AgraOBP1 | A0A2S1KLI2 | 2.53E-65 | 71.15 | Agra | TcasOBPC13 | D6X4H7 | 4.2E-09 | 27% | Tcas |
| **AeugOBP14** | Classic | 635 | 3′ (441) | 147aa | AgraOBP8 | A0A2P9JZE8 | 1.24E-69 | 73.68 | Agra | TcasOBP14 | D2A254 | 7.5E-18 | 34% | Tcas |
| **AeugOBP24** | Classic | 543 | (414) | 138aa | AgraOBP6 | A0A2P9JZE6 | 1.86E-79 | 83.94 | Agra | TcasOBP24 | D6WNH1 | 1.04E-44 | 51% | Tcas |
| **AeugOBP73a** | Classic | 458 | (459) | 153aa | ItypOBP12 | M3VHC4 | 1.24E-79 | 78.16 | Ityp | DmelOBP73 | Q7KUQ3 | 1.52E-20 | 47% | Dmel |

| | | | | | | | | | | | | | | |
|---|---|---|---|---|---|---|---|---|---|---|---|---|---|---|
| **AeugOBP83b** | Classic | 413 | I (411) | 137aa | - | I1VJ73 | 2.8 E-20 | 28.46 | Dpon | DmelOBP83b | Q23970 | 6.63E-07 | 27% | Dmel |

5′: 5-prime partial, 3′: 3-prime partial, I: incomplete, aa: number of amino acids, Acc. Num: Accession Number, %Id: Identity percentage, Sp: Species with best homology match. Species: *Anthonomus grandis* (Agra), *Dendroctonus ponderosae* (Dpon), *Ips typographus* (Ityp), *Rhynchophorus ferrugineus* (Rfer), *Tribolium castaneum* (Tcas), *Drosophila melanogaster* (Dmel).

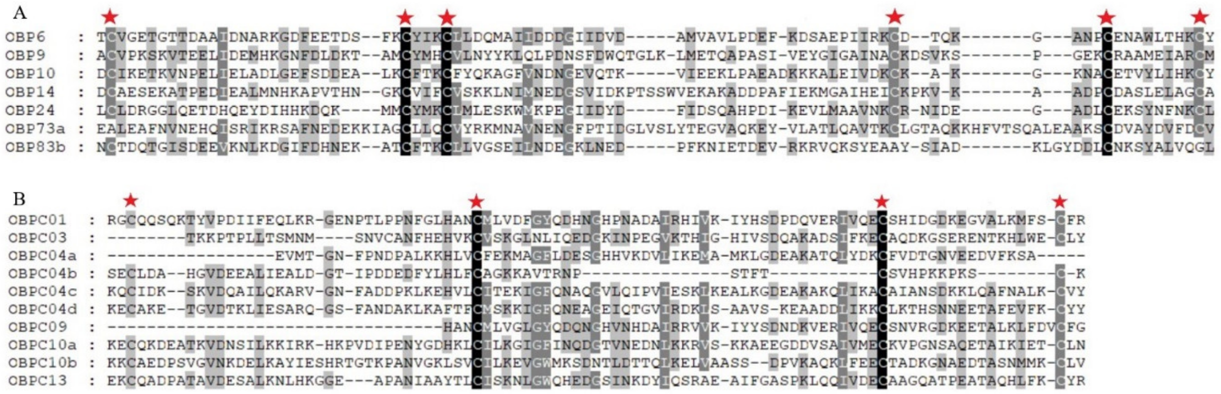


**Figure S1.** Multiple sequence alignments of AeugOBPs. A) Classic OBPs, with six conserved Cysteines (Cys). B) Minus-C OBPs, with four conserved Cys. Red marks indicate conserved Cys, the higher conservation of residues is depicted in darker colors.

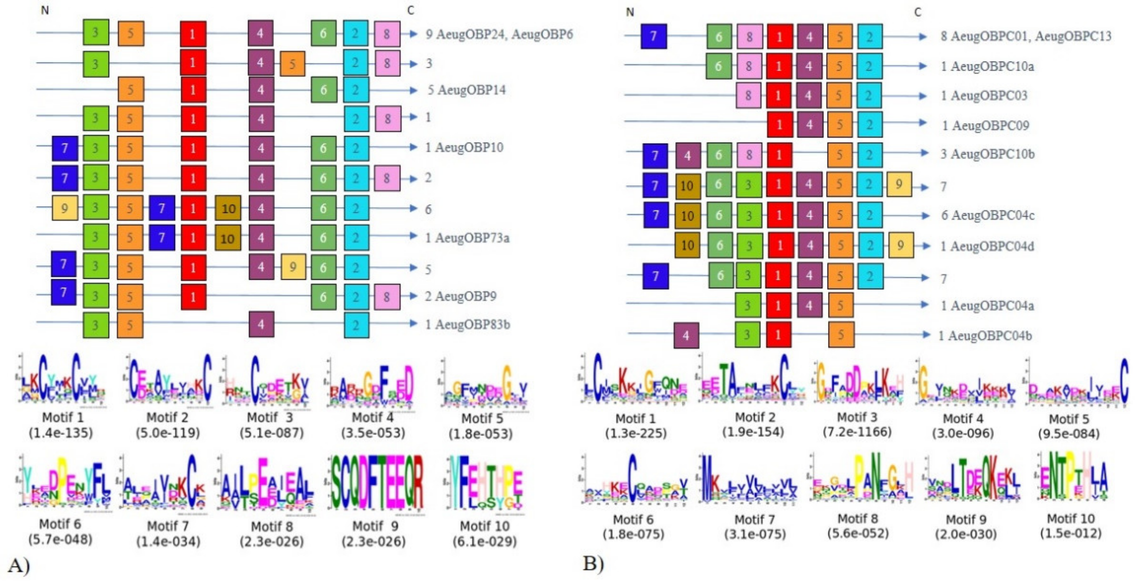


**Figure S2.** Motif architectures of AeugOBPs. Color squares represent each motif and the height of letters in the motifs represents the amino acid residue frequency. A) Classic OBPs motifs architectures. B) Minus-C OBPs motifs architecture. Num. OBP: the numbers at the right of each architecture represent the
sequences with similar architecture, including AeugOBPs.

**Table S5.** Results of UniProtKB and DescribePROT homology analysis. The table shows the best hits with the highest percentage of identity.

| AeugCSP | Unigen (bp) | ORF (bp) | Protein (aa) | Best BLASTx match (UniProtKB) | | | | | Best DescribePROT match | | | | |
|---|---|---|---|---|---|---|---|---|---|---|---|---|---|
| | | | | **Name** | **Acc- Num.** | **E-value** | **% Id** | **Sp.** | **Name** | **Acc- Num.** | **E-value** | **% Id** | **Sp.** |
| AeugCSP1 | 508 | 5′ (285) | 94 | AechCSP3 | F4WYC0 | 1.4 E-026 | 59.67 | Aech | CSP1/CSP7K | Q0MRL2 | 3.68E-26 | 52 | Tcas |
| AeugCSP1a | 798 | 5′ (420) | 140 | AgraCSP8 | AVI04879.1 | 2E-34 | 98.33 | Agra | CSP1/CSP7K | Q0MRL2 | 3.54E-24 | 78 | Tcas |
| AeugCSP10 | 531 | (378) | 125 | AgraCSP5 | A0A2P9JZD6 | 3.66E-61 | 85.04 | Agra | CSP10/CSP7J | Q0MRL3 | 6.7E-51 | 56 | Tcas |
| AeugCSP11 | 323 | I (321) | 106 | AgraCSP2 | A0A2P9JZD5 | 2.83 E-053 | 84.88 | Agra | CSP11/CSP7G | Q0MRL9 | 6.08E-38 | 73 | Tcas |
| AeugCSP13 | 209 | 3′ (201) | 66 | AgraCSP10 | AWF93846.1 | 1E-30 | 91.49 | Agra | CSP13 | Q0MRL7 | 4.23E-25 | 71 | Tcas |
| AeugCSP14 | 765 | (441) | 146 | AgraCSP1 | A0A2P9JZD4 | 6.94 E-68 | 76.69 | Agra | CSP14 | Q0MRL8 | 1.79E-44 | 56 | Tcas |

5′: 5-prime partial, I: incomplete, aa: number of amino acids, Acc. Num: Accession Number, %Id: Identity percentage, Sp: Species with best homology match. Species: *Anthonomus grandis* (Agra), *Dendroctonus ponderosae* (Dpon), *Tribolium castaneum* (Tcas), *Acromyrmex echinatior* (Aech).

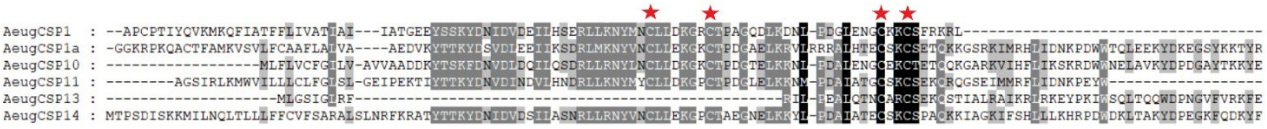

**Figure S3.** Multiple alignments of AeugCSPs. Red marks indicate conserved Cys, the higher conservation of residues is depicted in darker colors.

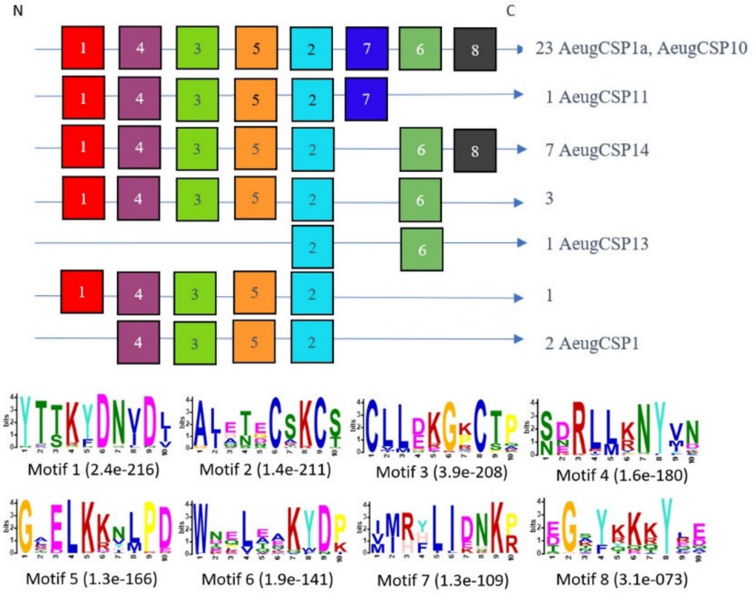


**Figure S4.** Motif architectures of AeugCSPs. Color squares represent each motif and the height of letters in the motifs represents the amino acid residue frequency. The numbers at the right of each architecture represent the sequences with similar architecture, including AeugCSPs.

**Table S6.** Accession numbers of Odorant-Binding Proteins and Chemosensory Proteins used in phylogenetic analysis

| OBP | | CSP | |
|---|---|---|---|
| **AchiOBP7** | AUF72991 | AgraCSP1 | AVI04873.1 |
| **AglaOBP16** | A0A1W5XGL5 | AgraCSP2 | AVI04874.1 |
| **AgraGOBP1** | AWF93844.1 | AgraCSP3 | AWF93845.1 |
| **AgraOBP1** | AWF93831.1 | AgraCSP4 | AVI04875.1 |
| **AgraOBP3** | A0A2P9JZE4 | AgraCSP5 | AVI04876.1 |
| **AgraOBP3_partial** | A0A2S1KLJ4 | AgraCSP7 | AVI04878.1 |
| **AgraOBP4** | AVI04885.1 | AgraCSP8 | AVI04879.1 |
| **AgraOBP5** | A0A2P9JZE5 | AgraCSP9 | AVI04880.1 |
| **AgraOBP6** | A0A2P9JZE6 | AgraCSP10 | AWF93846.1 |
| **AgraOBP7** | AVI04888.1 | AgraCSP11 | AVI04881.1 |
| **AgraOBP8** | A0A2P9JZE8 | AgraCSP12 | AVI04882.1 |
| **AgraOBP9** | A0A2P9JZE9 | DponCSP1 | AKK25146.1 |
| **AgraOBP10** | AVI04891.1 | DponCSP2 | AKK25147.1 |
| **AgraOBP11_partial** | AWF93840.1 | DponCSP3 | AGI05160.1 |
| **AgraOBP12** | A0A2P9JZF1 | DponCSP8 | AGI05164.1 |
| **AgraOBP13** | AVI04893.1 | DarmCSP1 | AXF54070.1 |

| | | | |
|---|---|---|---|
| **AgraOBP15** | AVI04895.1 | DarmCSP7 | AXF54074.1 |
| **AgraOBP16** | A0A2P9JZF4 | DarmCSP8 | AXF54075.1 |
| **AgraOBP18** | AVI04897.1 | DadjCSP1 | QPZ89235.1 |
| **AgraOBP20_partial** | AWF93842.1 | DadjCSP3 | QPZ89237.1 |
| **AgraOBP21** | AVI04899.1 | DadjCSP8 | A0A7T3R1R8 |
| **AgraOBP22** | AWF93843.1 | CbowCSP12 | ALR72526.1 |
| **AgraOBP23** | AVI04900.1 | LoryCSP6 | AHE13802.1 |
| **CforOBP3** | A0A6B7M5S2 | LoryCSP8 | AHE13803.1 |
| **CforOBP8** | A0A481VAY9 | TcasCSP1 | Q0MRL2 |
| **CforOBP18** | A0A6B7MBM4 | TcasCSP10 | Q0MRL3 |
| **ClapOBP17** | A0A6B7M084 | TcasCSP11 | Q0MRL9 |
| **DadjOBP8** | A0A7U3W596 | TcasCSP13 | Q0MRL7 |
| **DadjOBP11** | A0A7U3W597 | TcasCSP14 | Q0MR8 |
| **DadjOBP12** | A0A7U3RLR2 | TyunCSP1 | AMP19496.1 |
| **DadjOBP18** | A0A07U3W5A0 | TyunCSP2 | AMP19497.1 |
| **DadjOBP23** | A0A7U3U820 | TyunCSP4 | AMP19499.1 |
| **DadjOBP30** | A0A7U3UIM6 | AgraCSP1 | AVI04873.1 |
| **DadjOBPj74** | *Pedir Brenda | AgraCSP2 | AVI04874.1 |
| **DadjOBPj75** | A0A7U3UIM0 | AgraCSP3 | AWF93845.1 |
| **DarmOBP13** | A0A0X9FBE8 | AgraCSP4 | AVI04875.1 |
| **DponOBP3_1** | M4VR73 | AgraCSP5 | AVI04876.1 |
| **DponOBP4_2** | AKK25132.1 | AgraCSP7 | AVI04878.1 |
| **DponOBP7_2** | JAA74496.1 | AgraCSP8 | AVI04879.1 |
| **DponOBP8** | M4VRL6 | AgraCSP9 | AVI04880.1 |
| **DponOBP9** | M4VRM7 | AgraCSP10 | AWF93846.1 |
| **DponOBP9_partial** | A0A0H3W5N6 | AgraCSP11 | AVI04881.1 |
| **DpondOBP11** | M4W9C1 | AgraCSP12 | AVI04882.1 |
| **DponOBP13_2** | J3JVJ5 | DponCSP1 | AKK25146.1 |
| **DponOBP18** | AKK25142.1 | | |
| **DponOBP17_2** | AKK25141.1 | | |
| **DponOBP19_1** | AGI05183.1 | | |
| **DponOBP19 2** | A0A0H3W5L4 | | |
| **DponOBP28** | M4VUX0 | | |
| **DponOBP29** | AGI05182.1 | | |
| **DponOBP30** | M4W9B7 | | |
| **DponOBP56d** | J3JVJ5 | | |
| **DponOBP70** | J3JXC2 | | |
| **ItypOBP1_partial** | M3VK49 | | |
| **ItypOBP4** | M3TYY8 | | |
| **ItypOBP8_partial** | M3V873 | | |
| **ItypOBP7** | M3VHC0 | | |
| **ItypOBP12** | M3VHC4 | | |

| | |
|---|---|
| **ItypOBP14** | M3TYZ1 |
| **LoryOBP1_partial** | A0A0B4KZD3 |
| **LoryOBP14** | A0A0B4KZC6 |
| **RdomPBP3** | A0A0X8T3G6 |
| **RferOBP8** | A0A1C8JYW3 |
| **SzeaOBP8_partial** | A0A4V1GPX0 |
| **SzeaOBP21** | A0A4P9CZ87 |
| **SzeaOBP22** | A0A4P9CZ21 |
| **TcasOBPC04** | D6WS38 |
| **TcasOBPC05** | EFA07543.1 |
| **TcasOBP6** | D6WM82 |
| **TcasOBPC06** | D6WS47 |
| **TcasOBPC08** | EFA07638.1 |
| **TcasOBPC09_2** | D6WS40 |
| **TcasOBPC09_1** | D6X0T3 |
| **TcasOBP10** | D6WS37 |
| **TcasOBP9** | D6X1W1 |
| **TcasOBPC21** | EFA07491.1 |
| **TcasOBP24** | EFA04576.1 |
| **TcasGOBP56d** | D6WS37 |
| **TcasOBP70_X1** | XP_008194709.1 |
| **TcasOBP70_X2** | XP_008194712.1 |
| **TcasOBP72 2** | D6X1W1 |
| **TcasGOBP83a** | D6WM82 |
| **TyunOBP7** | A0A4P2HN13 |
| **TyunOBP8** | A0A4P2HMC9 |
| **TyunOBP9** | A0A4P2HMQ1 |
| **TyunOBP10** | A0A4P2HMJ8 |
| **TyunOBP11** | A0A4P2HSM5 |

**Table S7**. Primers used in genes amplification of AeugOBPs

| **Gen** | **Primer** | **5′-3′ Sequence** | **Amplicon size (bp)** | **Ta (°C)** |
|---|---|---|---|---|
| AeugOBPCO1 | OBPC01-F | GGTCGATGGGATTAGCACACA | 383 | 60.7 |
| | OBPC01-R | TGCGCTCAACTTGATCAGGAT | | |
| AeugOBPC04a | OBPC04a-F | GCAACTTTCCTAATGATCCGGC | 208 | 60.2 |
| | OBPC04a-R | GCACTCTTGAAAACGTCTTCCT | | |
| AeugOBPC04b | OBPC04b-F | CCTGACGACGAGGATTTCTACC | 300 | 59.3 |
| | OBPC04b-R | CAGCCAAAAATTACAGCCCATG | | |
| AeugOBPC04c | OBPC04c-F | TGAAGGTGTTTTTCGCTGCTG | 310 | 60.9 |
| | OBPC04c-R | CAGGCCTTGATCAGCTGTTTG | | |

| | | | | |
|---|---|---|---|---|
| AeugOBPC09 | OBPC09-F | CTGTATGCTAGTTGGTCTTGG | 157 | 57.1 |
| | OBPC09-R | AGCAGTCTCTTCCTTATCACC | | |
| AeugOBPC10a | OBPC10a-F | TGTCAGAAAGATGAGGCCACT | 237 | 55.7 |
| | OBPC10a-R | TTCCTGGAACTTTGCATTCC | | |
| AeugOBP9 | OBP9-F | TTTGCGGCTCTGGTATTGGT | 401 | 58.5 |
| | OBP9-R | TGTGGATCAAACTCGTACATGC | | |
| AeugOBP10 | OBP10-F | GTTTCCGCCATTTCATGCCAG | 407 | 59.5 |
| | OBP10-R | TTCTTCTTCTCTTCTTTAGCCGC | | |
| AeugOBP14 | OBP14-F | GATGAATCACAAGGCACCAGT | 242 | 59.7 |
| | OBP14-R | CACATCCAGCAAGTTCTAGGC | | |
| AeugOBP24 | OBP24-F | GAAATTCCACCAGAGCTCCA | 250 | 54.8 |
| | OBP24-R | ATTTATTCACCGCAGCCATC | | |
| AeugOBP73a | OBP73a-F | GGAATTCCTCGTTCATGGGGA | 438 | 60.1 |
| | OBP73a-R | GCAACTTCTTCAGATACACAGTCG | | |
| AeugOBP83b | OBP83b-F | TGTGCTTATGGGATTCTGGCT | 305 | 60.1 |
| | OBP83b-R | GCAATGGAATATGCCGCTTCA | | |

Ta= Annealing temperature.

**Table S8**. Primers used in genes amplification of AeugCSPs.

| Gen | Primer | 5′-3′ Sequence | Amplicon size (bp) | Ta (°C) |
|---|---|---|---|---|
| AeugCSP10 | CSP10-F | CGTATTTCTTAGTGTAAGCGCCA | 301 | 59.1 |
| | CSP10-R | GGCCGCAGATGACAAATACAC | | |
| AeugCSP11 | CSP11-F | TCTGATCCTTGACGTTGCTTT | 275 | 59 |
| | CSP11-R | TGCTTATTTGGACTATCCCTTGGA | | |
| AeugCSP14 | CSP14-F | GGCTTTGTCGTTAAACCGGTT | 296 | 61.2 |
| | CSP14-R | TCGGGATCGTATTTCGCAGTC | | |

Ta= Annealing temperature.
